# The chromosome-scale genome assembly of cluster bean provides molecular insight into edible gum (galactomannan) biosynthesis family genes

**DOI:** 10.1038/s41598-023-33762-3

**Published:** 2023-06-19

**Authors:** Kishor Gaikwad, Goriparthi Ramakrishna, Harsha Srivastava, Swati Saxena, Tanvi Kaila, Anshika Tyagi, Priya Sharma, Sandhya Sharma, R. Sharma, H. R. Mahla, Kuldeep Kumar, Amitha Mithra SV, Amolkumar U. Solanke, Pritam Kalia, A. R. Rao, Anil Rai, T. R. Sharma, N. K. Singh

**Affiliations:** 1 ICAR-National Institute for Plant Biotechnology, New Delhi, India; 2 ICAR-Central Arid Zone Research Institute, Jodhpur, India; 3 Division of Vegetable Sciences, ICAR-Indian Agricultural Research Institute, New Delhi, India; 4 ICAR-Indian Agricultural Statistics Research Institute, New Delhi, India; 5 DDG (CS), Indian Council of Agricultural Research, New Delhi, India

**Keywords:** Biotechnology, Computational biology and bioinformatics, Molecular biology, Plant sciences

## Abstract

Cluster bean (*Cyamopsis tetragonoloba* (L.) Taub 2n = 14, is commonly known as Guar. Apart from being a vegetable crop, it is an abundant source of a natural hetero-polysaccharide called guar gum or galactomannan. Here, we are reporting a chromosome-scale reference genome assembly of a popular cluster bean cultivar RGC-936, by combining sequencing data from Illumina, 10X Genomics, Oxford Nanopore technologies. An initial assembly of 1580 scaffolds with an N50 value of 7.12 Mb was generated and these scaffolds were anchored to a high density SNP linkage map. Finally, a genome assembly of 550.31 Mb (94% of the estimated genome size of ~ 580 Mb (through flow cytometry) with 58 scaffolds was obtained, including 7 super scaffolds with a very high N50 value of 78.27 Mb. Phylogenetic analysis using single copy orthologs among 12 angiosperms showed that cluster bean shared a common ancestor with other legumes 80.6 MYA. No evidence of recent whole genome duplication event in cluster bean was found in our analysis. Further comparative transcriptomics analyses revealed pod-specific up-regulation of genes encoding enzymes involved in galactomannan biosynthesis. The high-quality chromosome-scale cluster bean genome assembly will facilitate understanding of the molecular basis of galactomannan biosynthesis and aid in genomics-assisted improvement of cluster bean.

Cluster bean (*Cyamopsis tetragonoloba* (L.) Taub.), also known as guar^1^ is a member of Leguminosae family. The common name cluster bean is attributed to its pods which appear in clusters. Previous reports suggest that it originated in Africa and later spread to the entire South Asian region. In India and Pakistan, cluster bean is cultivated since ancient times for its tender pods which are used as fresh vegetable and the remaining plant serves as fodder^2^. Cluster bean is a climate-resilient annual legume and a high potential alternative crop in the marginal lands of arid and semi-arid regions^3^. The genus *Cyamopsis* includes four species i.e., one cultivated *C. tetragonoloba* (L.) Taub., two wild relatives *C. serrata* Schinz, and *C. senegalensis * Guill & Perr, and *C. dentate* Tarre, an interspecies hybrid of *C. serrata and C. senegalensis*^4^.

A mature cluster bean seed is composed of three parts: germ (43–47%), endosperm (35–42%), and seed coat (14–17%). About 80–90% of the endosperm is composed of highly viscous water-soluble hetero-polysaccharide called guar gum (or) galactomannan, having a 1:2 composition of galactose to mannose^5^. Guar gum is extensively utilized as natural thickener, emulsifier and stabilizer in the food, textile, paper, petroleum and pharmaceutical industries with increasing global demand^6–9^. With the annual production of ~ 1–1.25 million tons of cluster bean seeds, India accounts for 80% of the global production, with several other countries, like Pakistan, United States, China, Australia and Africa contributing the rest. About 45% of total world demand is due to industrial application of guar gum^10^. USA is the largest importer of guar gum (244829 metric ton) from India, representing about 60% of the total followed by China (32268 metric ton) and Germany (12085 metric ton), which accounts for 7.8% and 2.9%, respectively (Guar gum market report, 2019)^11^. Apart from being a rich source of commercial product like gum, cluster bean is also a highly nutritious legume crop, predominantly composed of protein (18%) and dietary fiber (32%).

Earlier cytogenetic studies in cluster bean revealed that ~ 580.9 Mb of the genome is organized in 2n = 14 number of chromosomes^12,13^. Despite its considerable industrial importance, only a few studies have been carried out at genome level which includes genome size estimation (cultivated vs. wild type)^13^, chloroplast genome sequencing^14^, transcriptome analysis^15,16^, small RNA sequencing^17^ to identify novel miRNA associated with galactomannan biosynthesis as well as genetic diversity analysis based on SSRs^18^, mostly from our group. Therefore, it was necessary to sequence a chromosome-scale high-quality reference genome to understand the molecular basis of galactomannan biosynthesis, synteny with other legumes and discovery of genes for other important traits. This will also encourage cluster bean genetic improvement via genomics assisted breeding.

## Results

### Genome assembly and annotation

**Figure 1:**
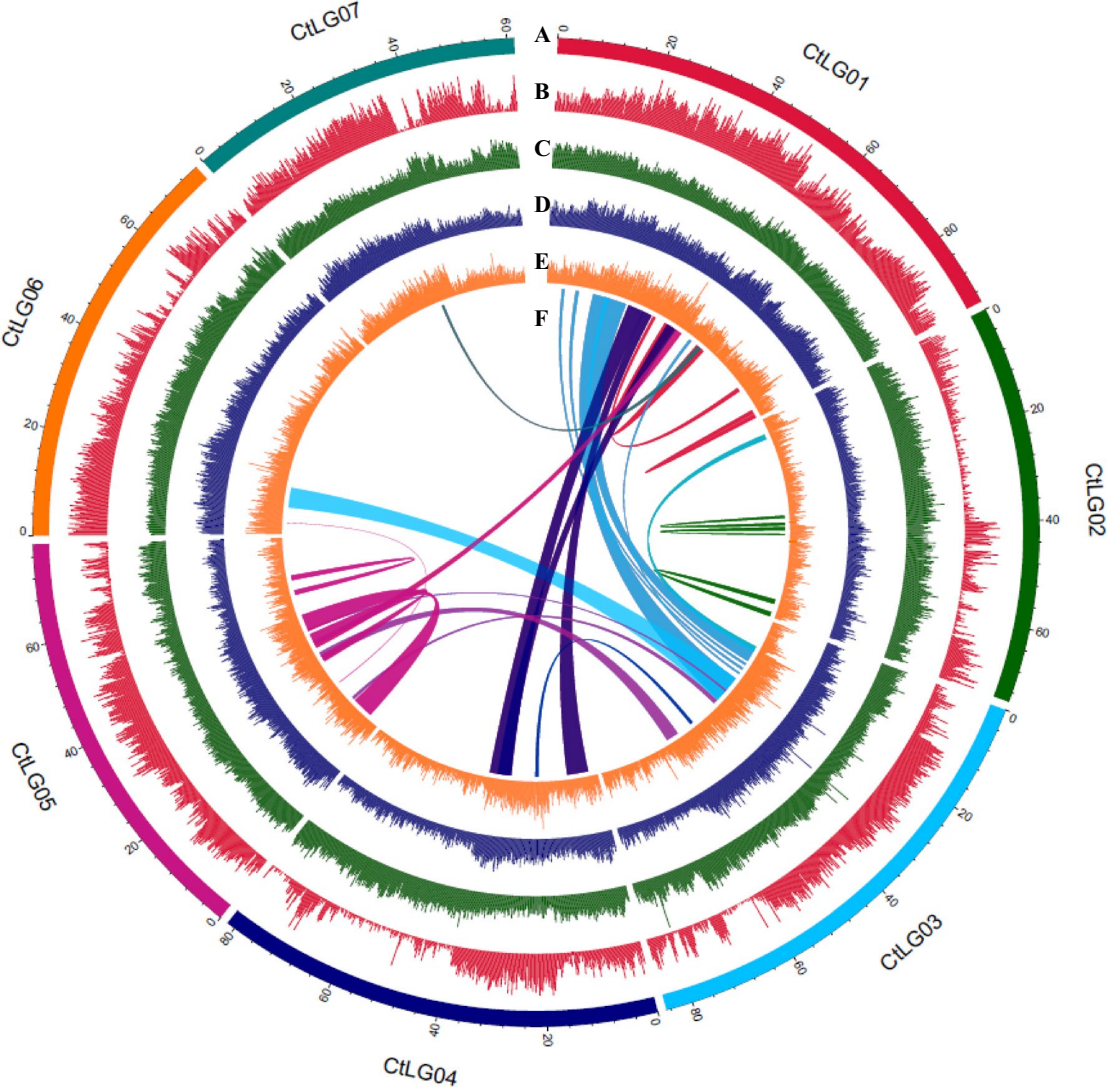
The cluster bean genome features. (**A**) Circular representation of the 7 pseudomolecules; (**B**) Genome wide distribution of protein coding genes; (**C**) Genome wide distribution of Retrotransposons; (**D**) Genome wide distribution of DNA elements; (**E**) Genome wide distribution of SSRs; (**F**) Distribution of syntenic regions in cluster bean genome.

A popular high yielding and galactomannan rich cluster bean cultivar RGC-936 was selected for whole genome sequencing and de novo assembly. We generated a total of 201.8 Gb raw sequencing data from various sequencing platforms corresponding to 366.73X genomic coverage of cluster bean genome. The cluster bean genome was de novo assembled by following hybrid assembly approach including 10X genomics linked read assembly from supernova and long read assembly from Canu with Oxford nanopore PromethION sequencing data. It was further improved by adding pair-end and mate-paired short read sequencing data resulting in 1580 scaffolds spanning 550.16 Mb with an N50 value of 7.12 Mb, with the longest scaffold size of 35.03 Mb. The assembled scaffolds were further anchored to genetic map with 7 linkage groups. Thus, the generated final assembly consists of 58 scaffolds covering 550.31 Mb of total length, and with 549.23 Mb (99.8%) of the assembled genome anchoring to 7 pseudomolecules with size ranging from 61.32 to 93.95 Mb and N50 (pseudomolecule) value of 78.27 Mb with very small number of gaps (N’s accounting for 0.27%). The final chromo-scale assembly size of 549.23 Mb is slightly (1.1%) higher than the estimated genome size of 543.22 Mb obtained from k-*mer* distribution analysis and 94.69% of the 580 Mb genome size estimated through Flow cytometry analysis. The detailed assembly statistics are summarized in Table 1 and Fig. 1.

**Table 1:** Cluster bean genome assembly, gene annotation and non-protein coding genes.

Cluster bean genome assembly features	
Total length of the assembly (bp)	550,317,005
GC content (%)	32.64
Number of scaffolds	58
Scaffold N50 (bp)	78,271,499
Longest scaffold (bp)	93,956,920
Number of contigs	1580
Max contig length (bp)	35,034,496
Min contig length (bp)	970
Contig N50 (bp)	7,127,297
Gene models	
Number of gene models	34,680
Mean transcript length (bp)	3823.77
Mean coding sequence length (bp)	289.115
Mean number of exons per gene	5.35043
Mean exon length (bp)	249.843
Mean intron length (bp)	681.044
Number of genes annotated	28,955
Number of genes unannotated	5725
Non-protein coding genes	
Number of rRNA fragments	922
Mean length of rRNA fragments (bp)	709.4143167
Percentage of rRNA fragments in genome	0.118855132
Number of tRNA genes	474
Mean length of tRNA genes (bp)	70.58
Percentage of tRNA genes in genome	0.006454462
Number of snRNA genes	347
Mean length of snRNA genes (bp)	100.39
Percentage of snRNA genes in genome	0.006330533
Total number of repeat elements	582,339
Total repetitive DNA length (bp)	231,879,649
Percentage of repetitive DNA in genome	42.13565034

Repetitive sequences account for 42.14% of the cluster bean genome of which 29.73% were retrotransposons (Table 2). As is the case of most plant genomes, the predominant type of transposable elements (TEs) was long terminal repeat (LTR), which accounts for 21.62% of the cluster bean genome, including 14.29% of LTR/Gypsy and 2.83% of LTR/Copia elements (Table 2 and Supplementary Table S7). The distribution of TEs was observed to be inversely correlated to gene density along the chromosomes (Fig. 1). After identification of repetitive sequences, the masked genome assembly was further used for gene prediction by Seqping2. A total of 34680 protein coding genes were predicted from cluster bean genome, of which 28955 (78.93%) were successfully annotated. Of these, 22325, 20853, and 23382 genes were involved in cellular functions, molecular functions, and biological processes respectively (Supplementary Fig. S6). In addition, a total of 922 rRNAs, 474 tRNAs and 347 snRNAs were identified in the cluster bean genome (Supplementary Fig. S7).

**Table 2:** Organization of repetitive sequences in the Cluster bean genome.

	Number	Length occupied (bp)	Total repeats (%)	Genome (%)
Total Retrotransposons	378584	163,602,743	70.55	29.73
LINE	155598	44,439,166	19.16	8.08
SINE	1100	176,409	0.08	0.03
LTR retrotransposons	221886	118,987,168	51.31	21.62
Gypsy	105335	78,656,908	33.92	14.29
Copia	27099	15,596,297	6.72	2.83
DNA transposons	185613	54,034,406	23.30	9.82
Unclassified elements	13555	12,889,753	5.56	2.34
Satellites	1611	412,566	0.18	0.07
ncRNA	1332	210,855	0.09	0.04
Total repetitive elements	582339	231,879,649	100.00	42.14

**Figure 2:**
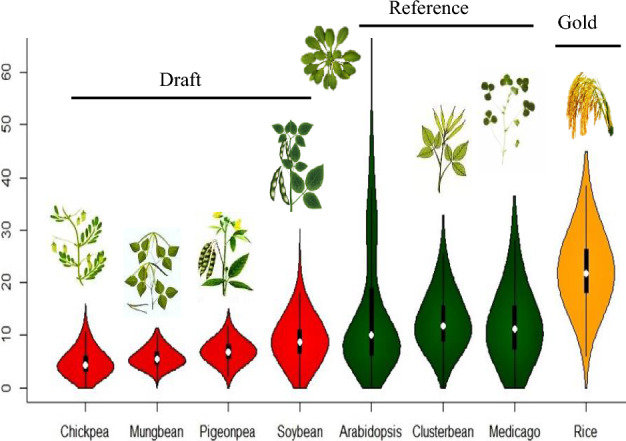
The LAI evaluation of the genome assembly of Cluster bean and seven other plant species including four grain legumes (Chickpea, Mungbean, Pigeonpea, and Soybean), two model plants (*Arabidopsis, Medicago*) along with a gold standard genome assembly of Rice.

Further the high-quality of the cluster bean genome assembly is evident from high LTR assembly index (LAI) and high Benchmarking Universal Single-Copy Orthologs (BUSCO) score. Long terminal repeat (LTR) annotation in the cluster bean revealed a LAI score of 11.04, which meets the standards of reference quality assembly, and is higher than any other crop legume used in current study (Fig. 2 and Supplementary Table S4). Identification of 1332 (96.9%) complete orthologs based on the BUSCO analysis further confirms the high-quality, continuity and completedness of cluster bean genome assembly. Further, we also found that 33% (452) of the complete BUSCO genes are duplicated (Supplementary Fig. S5).

### Comparative genomic and phylogenetic analysis

Ancient whole genome duplication (WGD) or polyploidization is an important force of evolution of all the organisms, including animals, fungi and particularly plants^19,20^. The high-quality reference genome of cluster bean enabled us to perform comparative genomics among some of the representative angiosperms. We selected a range of legume species to investigate WGD in cluster bean through comparative genomics.

**Figure 3:**
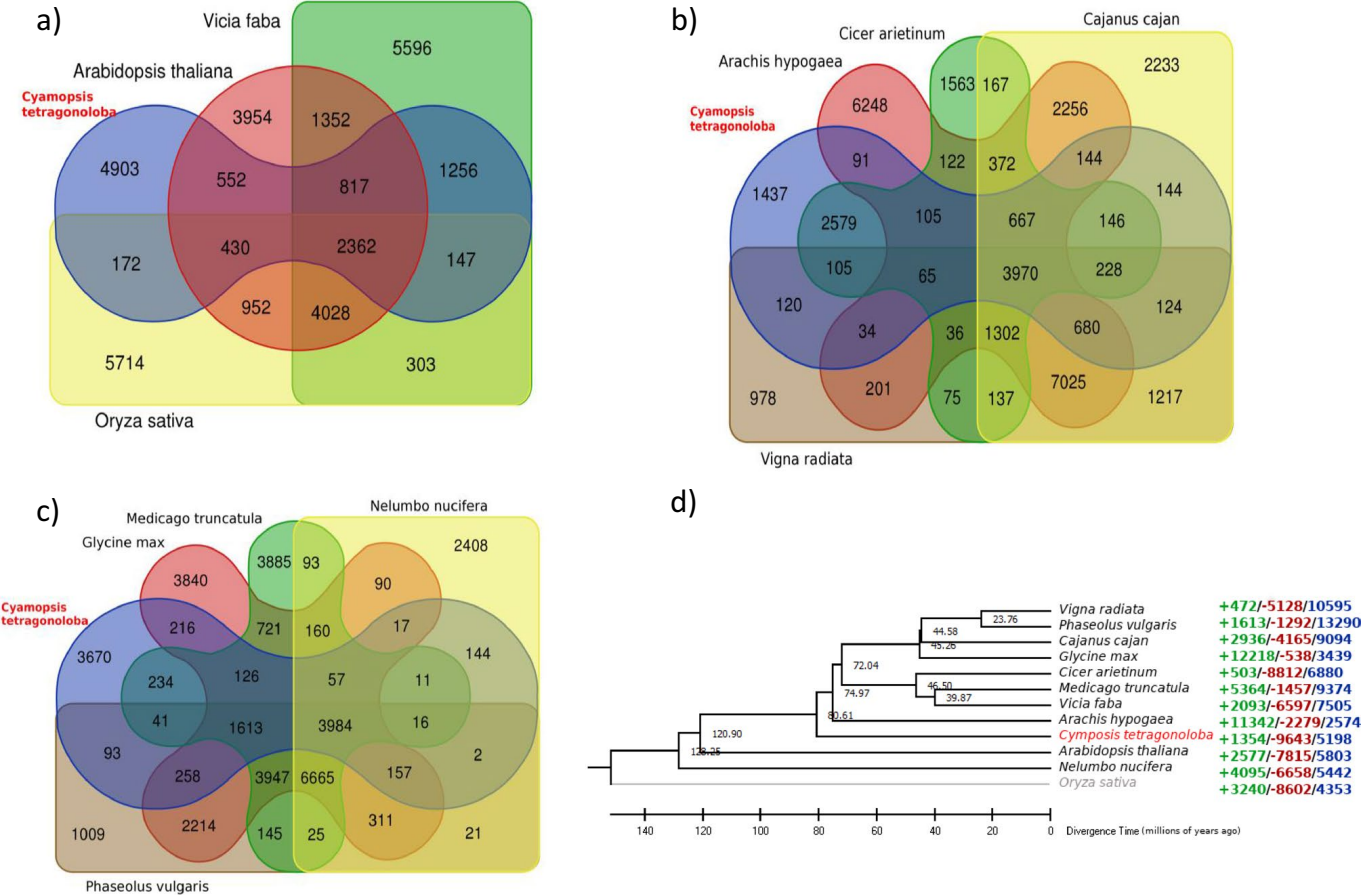
Gene family analysis of predicted cluster bean genes in comparison to major crop and model plant genomes and phylogenetic analysis of the same with 51 common Orthologs and the number of gene families experienced gene expansion (Green), Gene loss (Red), and Gene retained (Blue).

To understand the evolutionary history of cluster bean genome, we conducted a gene family clustering analysis using cluster bean and 11 other representative angiosperm species including model plant. These include 8 species from Fabaceae family (*Phaseolus vulgaris, Vigna radiata, Cajanus cajan, Glycine max, Cicer arietinum, Medicago truncatula, Vicia faba, Arachis hypogaea*), Two from Eudicot clade (*Arabidopsis thaliana* and *Nelumbo nucifera*) and one outgroup species (*Oryza sativa*). We identified 51 common single copy orthologs and used them for phylogenetic tree construction and species divergence time estimation. Our results indicated that cluster bean and other studied legumes may have shared a common ancestor ~ 80.61 million years ago (MYA) (Fig. 3).

We tried to understand the genomic basis of galactomannan biosynthesis. We used OrthoMCL to identify orthologous genes and gene families and CAFÉ tool to identify the gene families showing expansion (gain) and contraction (loss) during evolution with specific focus on galactomannan biosynthesis genes in cluster bean. During this process we identified 193721 gene families consisting of 306710 genes among 11 species (Supplementary Table S9). The results of CAFÉ analysis showed that 1354 gene families experienced expansion while 9643 gene families faced gene loss and 5198 gene families remained unchanged during evolution (Fig. 3). The major gene families experiencing expansion were UDP-glucuronate 4-epimerases, Glycosyl transferases, and CLIP-associated protein-like. The Glycosyl transferases are the main class of enzymes which play important role in galactomannan biosynthesis.

### Whole genome duplication

**Figure 4:**
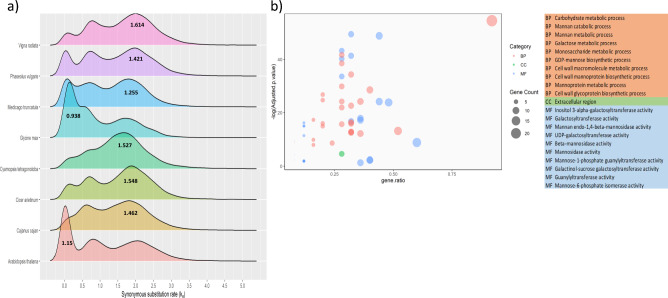
(**a**) Frequency distributions of synonymous substitution rates (Ks) between paralogous genes of Cluster bean *(Cyamopsis tetragonoloba), Vigna radiata, Phaseolus vulgaris, Medicago truncatula, Glycine max, Cicer arietinum, Cajanus cajan, and Arabidopsis thaliana.* (**b**) Enrichment analysis of genes retained after the ancient WGD of Cluster bean genome.

The analysis of cluster bean genome with WGDdetector for the identification of whole genome duplication (WGD) event resulted in the identification of 13700 paralogous genes from a total 5094 homologous gene clusters, retained post-WGD. Further, the self-synteny analysis of cluster bean genome assembly with SyMAP identified 13777 paralogous genes corresponding to 52 inter and intrachromosomal syntenic blocks ranging from 56.09 kb to 10.49 Mb. Further, synonymous substitution rates were calculated for all the paralogous genes (Fig. 4 and Supplementary Table S11).

**Figure 5:**
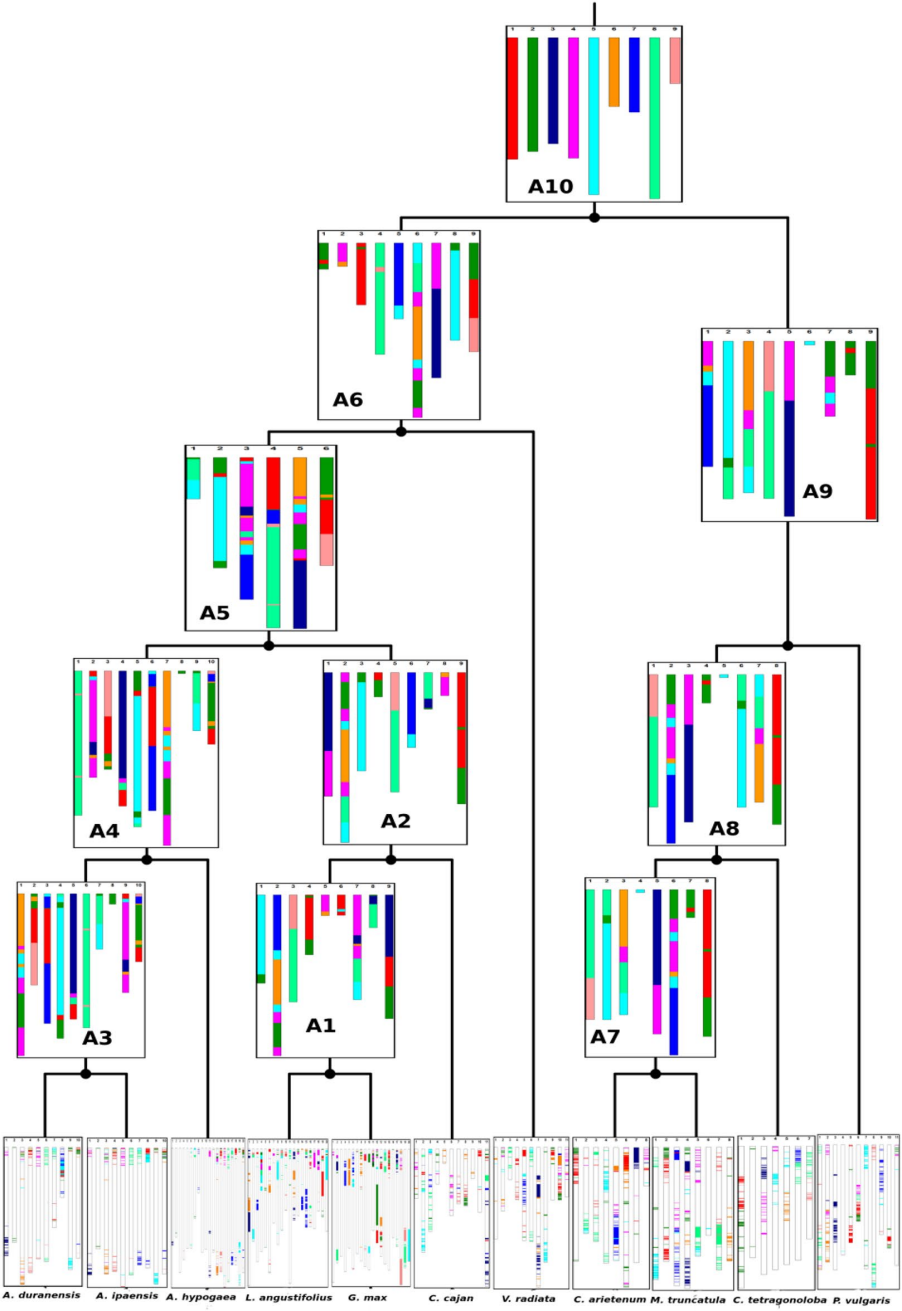
Evolution of legume genomes. Reconstructed ancestral genomes (A1-A10) and genomes of selected legumes are represented in a tree lay out and colour of the chromosomal segments are linked to source chromosome in the ancestral genome.

Further to investigate the evolution of cluster bean genome and other legume, we analyzed 235 syntenic blocks among the selected 11 legume species. The current interpretation of our results indicated that the common ancestor may have contained nine chromosomes (Fig. 5).

### Transcriptome analysis

**Figure 6:**
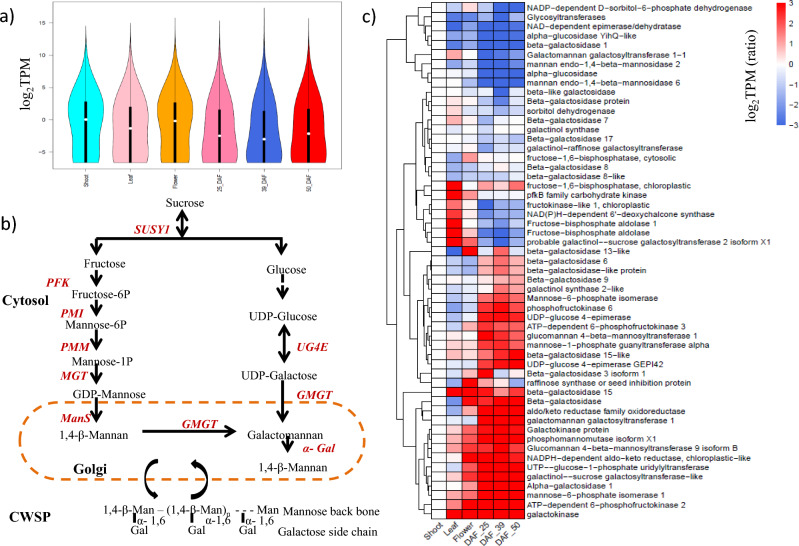
Transcriptome analysis of six cluster bean tissues (Shoot, Leaf, Flower, Pod_25_DAF, Pod_39_DAF, and Pod_50_DAF). (**a**) Violin plot showing transcript abundance in six cluster bean tissues. (**b**) Schematic representation of galactomannan biosynthesis pathway. (**c**) Heat map showing expression pattern of some of the genes involved in galactomannan biosynthesis among six cluster bean tissues.

In the present study, we conducted transcriptome analysis of RNA-Seq data from six different tissues to elucidate galactomannan biosynthesis. Galactomannan is synthesized from sucrose precursor in a multi-step reaction catalyzed by three major glycosyl transferase enzymes viz*.* Sucrose synthase (*SUS 1*), Mannan synthase (*ManS*) and Galactomannan galactosyl transferase (*GMGT*). In the first step *SUS 1* catalyzes reversible breakdown of sucrose to fructose and glucose. Later, fructose is converted into 1,4-β-Mannan and glucose is converted into UDP-Galactose in two separate multi-step reactions involving isomerases and epimerases enzymes respectively. Finally, *GMGT* catalyzes the synthesis of galactomannan which is deposited on the plant cell wall (Fig. 6).

The reference-based transcriptome analysis of six different tissues resulted in identification of 68641 transcripts. All the transcripts identified were annotated using Blast2GO and further KEGG pathway analysis was carried out to identify genes involved in galactose and mannose metabolism. The results indicate that pod-specific up-regulation of genes encoding Phosphofructokinase (PFK), Mannose-6-phosphate isomerase (PMI), Phosphomannomutase (PMM), Mannose-1-phosphate guanyl transferase (MGT), Glucomannan 4-beta- mannosyl transferase (ManS) which serially process the conversion of fructose into 1,4-beta mannan. Further genes involved in the conversion process of glucose into UDP-Galactose, such as UTP-Glucose-1-phosphate uridyltransferase, UDP-Glucose-4-epimerase (UG4E) were also up-regulated during different stages of pod development. Galactomannan galactosyltransferase (GMGT), the enzyme that catalyzes the final step during galactomannan biosynthesis is also up-regulated in pod tissues. The expression values (log2 fold change) of some of the differentially expressed genes in six tissues are depicted in Fig. 6. The results showed pod-specific up-regulation of genes encoding enzymes involved in galactomannan biosynthesis.

## Discussion

The Fabaceae or Leguminosae family comprises 751 genera and 19,500 species and represents the third largest family of flowering plants^21^. Legumes are rich source of protein for human and animal consumption apart from some commercially important products like gums (galactomannan) and dyes and these species also have the ability to fix atmospheric nitrogen thereby boosting soil fertility. The biosynthesis of mannose and galactose is a common metabolic pathway occurring in most plants, but very few plant species such as Fenugreek, Locust bean, Tamarind, *Cassia* sp., and Cluster bean can produce galactomannans. However, the ratios of mannose to galactose units varies from 1:3.75 to 5:1 in these plants^22^, and the information on regulation of genes encoding key enzymes involved in gum production remain unclear. Thus their divergence in Cluster bean and other gum producing crops needs to be analyzed for better harvest and extraction of this edible commodity.

The “-omics” era has provided a new set of tools and methods that have a significant impact on metabolic engineering and synthetic biology^23^. This has accelerated the search of genes and enzymes of specialized pathways and have aided in production of the compounds of interest by designing of the metabolic pathways^24,25^. The synthetically produced galactomannosides which mimic the galactomannan of *Aspergillus* were observed to trigger the immunological responses^24^. Thus identification of such novel pathways can lead to varied applications across different metabolic pathways for the benefit of human and animal consumption.

Acknowledging the challenges in genome assembly of highly repetitive and complex plant genomes, we followed a hybrid assembly approach for developing high-quality chromosome-level genome of cluster bean. The third-generation sequencing platforms like Oxford Nanopore sequencing provide long reads to facilitate the continuous sequence coverage in the repeat-rich regions and ensuring the generation of longer scaffolds during genome assembly. Additionally, genetic linkage maps based on recombination rates between physical markers have been used in biology for over 100 years and when paired with a de novo sequencing project, can resolve mis-assemblies and anchor chromosome-scale sequences. In the present study, a preliminary hybrid assembly from 10X Genomics and ONT was generated to 550.16 Mb consisting of 1580 scaffolds which were anchored in to 7 super scaffolds or pseudomolecules using a genetic map generated by using GBS of 142 F_2_ progenies and two parental genotypes.

In our analysis, the SNPs were distributed randomly across the chromosomes, with variable densities. The same was observed for the SSR markers identified in the current study. The cluster bean genome contained 238,176 SSRs, and demonstrated utility for diversity analysis of 54 cluster bean cultivars and 2 wild relatives^18^ thus confirming the value of the assembled genome.

A stringent threshold was set for filtering the SNPs, reducing the SNP density on every chromosome, after which chromosome 2 was under represented. We understand that inaccuracies in genetic maps can result from genotyping errors, but the limited number of informative meiosis needed to generate maps is the major limiting factor, which have been also reported previously^26^, which could be the reason of missing intervals on different linkage groups in our case. Errors in the order of markers on physical maps might arise due to problems with assembly or incorrect identification of marker positions^27,28^, but even if the order of markers is known to be without error, accurate estimates of recombination fractions will play an important role in linkage, which in turn depends on the number of cross over events^29^, further limited by number of progenies considered. The SSR makers present across all chromosomes can be utilized further to develop high density linkage map in order to curtail this discrepancy in advanced mapping population like RILs.

The final assembly reported here is the first ever high-quality chromosome-scale assembly of cluster bean genome corresponding to 7 chromosomes (haploid chromosome number). The size of the final assembly covered 550.31 Mb (94.73%) of the estimated genome size of 580 Mb based on the flow cytometry analysis^13^. This final assembly is one of the few near complete genome assemblies among plants with 549.23 Mb (99.8%) of the 550.31 Mb of assembled genome anchoring to 7 pseudomolecules with very high N50 (pseudomolecules) value of 78.27 Mb. Further, cluster bean genome assembly has a LAI score of 11.04 which is highest among the major grain legumes, thus validating our entire assembly approach. Moreover, the transcriptome data that was assembled using this genome indicated the stage and tissue specific expression of the key genes of the galactomannan synthesis pathway like Glucomannan 4-beta- mannosyl transferase (ManS) and Galactomannan galactosyltransferase. Our comparative studies have also revealed expansion of some of these major gene families indicating their evolutionary significance. Together, it can be assumed that Cluster bean probably diverged from the ancient legume ancestor and the expansion of galactomannan producing pathway aided in evolution and wider adaptability especially across the arid regions of the Indian subcontinent. Probably, the high content of the galactomannans help the seed survive extreme high temperatures and desiccation which gives this crop better survivability and hardiness. We now believe that these are interesting candidates for gene targeting assays for increasing both production and quality (galactose:mannan) of the Cluster bean galactomannans owing to its wider industrial applications.

While phylogenetic analysis using the conserved plastid based genes has dominated but with the advent of the sequencing technologies, it has been overtaken by nuclear genes^30^. Nuclear genes comparatively have slower rate of evolution and therefore contains more information when our objective is to distinguish closely related species^31^. During evolution speciation leads to development of orthologous genes while duplication leads to development of paralogous genes. Not all paralogs evolve at similar rates, therefore it is more imperative to use single copy orthologs for establishing the species level phylogenetic tree^32,33^. Parallelly, while constituting an ancestral genome we are more focused on tracing the structural evolutionary histories of those genomes Also, in structural evolution we compare the conservation of the syntenic block among the selected genomes^34^. In genome reconstruction species are placed in proximities based on the possible estimated recombination between them^35^. In ancestral genome construction, syntenic blocks are made between the conserved gene orthologs (percent similarity not compared). Gene duplication, segmentation duplication etc., are also considered i.e. it consider the syntenic blocks between both orthologs as well paralogs genes. In our case the difference in positioning of the *C. tetragonaloba* in phylogenic analysis as well as ancestral genome possible have arisen due to this principal differences between both methodologies.

The present report of cluster bean genome assembly is the first among the galactomannan (gum) producing plants; the comparative transcriptome analysis of six different tissues has improved our understanding of galactomannan biosynthesis. Therefore, our study provides valuable insights that may serve as foundation for future research on cluster bean improvement.

## Materials and methods

### Plant material

Seeds of the pure homozygous cluster bean variety, ‘RGC-936’ were obtained from ICAR-IARI and ICAR-CAZRI and maintained at ICAR-NIPB, New Delhi, India using the standard crop management practices. Leaf samples were collected from healthy plants and snap frozen in liquid nitrogen and stored at −80 °C till further use. The genomic DNA was extracted using CTAB method^36^. The integrity of DNA was tested by separating the DNA on a 0.8% agarose gel and the quantity of DNA was checked using DeNovix DS-11 spectrophotometer. The high-quality DNA was used for genome sequencing by Illumina and HMW DNA was used for 10X Genomics and Oxford Nanopore sequencing. Similarly, leaf samples of a F_2_ population (RGC 936 × CAZRI-15-3-8) were processed for GBS sequencing (Illumina).

### Genome sequencing

In the present study, we selected both short and long-read sequencing methods such as Illumina, 10X Genomics and Oxford Nanopore sequencing technology (ONT) for cluster bean genome sequencing.

For Illumina short-read sequencing, high-quality genomic DNA was randomly fragmented by the M220 Focused-ultra sonicator system (Covaris Inc, USA). Two genomic DNA libraries of 500–1000 bp insert size were prepared using the TruSeq DNA PCR-Free Sample Preparation Kit, as per the manufacturer’s guidelines. To realize sequence variation and high genome coverage (length), two separate Mate-Pair (MP) libraries of 3 kb and 7 kb insert size were prepared using Nextera Mate-Pair Sample Preparation Kit (Illumina, San Diego, CA). Both the Illumina PE and MP libraries were sequenced on Illumina HiSeqX Ten platform and produced 16.09 Gb and 42.3 Gb of (2X150 bp) sequencing data respectively (Supplementary Table S1).

For 10X Genomics sequencing, a total of 8 Gemcode libraries were prepared from high-quality DNA fragments longer than 50 kb, using the Chromium instrument (Chromium Genome v1, PN-120229). Sequencing of these libraries was performed on Illumina, HiSeq X Ten platform, generating 2 × 150 bp reads, resulting in a total of 92.767 Gb of 10X Genomics linked read raw sequencing data (Supplementary Table  S1).

For long-read Nanopore sequencing, genomic DNA was size-selected using BluePippin BLF7510 cassette (Sage Science) and high-pass mode (> 20 kb) and library was prepared using Oxford Nanopore Technologies (ONT) standard ligation sequencing kit SQK-LSK109 following the SQK-MAP005 PromethION protocol. Two libraries were prepared and sequenced using PromethION. A total of 50.64 Gb of Nanopore sequencing data was generated.

As a result, we generated a total of 201.8 Gb raw sequencing data corresponding to 366.73X genomic coverage (depth), of cluster bean genome (543.22 Mb estimated genome size using *k*-mer frequency distribution analysis). Details of sequencing data are represented in Supplementary Table S1.

### Genome size estimation

As per the earlier studies from our group, the genome size of cultivated guar (RGC-936) along with two crop wild relative species i.e., *C. serrata* and *C. senegalensis*, was estimated to be 580.9 Mb ± 0.02 (1C), 979.6 Mb ± 0.02 (1C), and 943.4 Mb ± 0.03 (1C) respectively^13^. The 31-mer abundance was calculated using Jellyfish v2^37^ and the cluster bean genome size was then estimated using Genomescope software^38^ (http://qb.cshl.edu/genomescope/genomescope2.0/). Total 1,187,123,081 k-mers (31-mer) were counted and their frequency distributions were analyzed. In the 31-mer frequency distribution histogram, the main peak was observed at depth of ~ 67 corresponding to homozygous haploid sequences. A small peak was also observed at half of the main peak depth representing heterozygous fraction of the genome (Supplementary Fig. S1). Thus, the cluster bean genome size was estimated to be 543.2 Mb (543,218,592 bp), and the fraction of heterozygosity in cluster bean genome was estimated to be in the range of 0.70–0.71%.

### Genome assembly and evaluation

We followed a hybrid assembly approach for developing high-quality chromosome-level genome of cluster bean. Initially, we generated two separate de novo assemblies by using 10X genomics linked reads and Oxford Nanopore long reads. We then merged both the assemblies to get minimal sequencing errors and a high degree of sequence continuity for scaffolds.

The raw 10X genomics sequencing data was assembled using Supernova (v2.1.1)^39^, which generates raw assembly as well as scaffold assembly. Removal of vector and mitochondrial contamination was done using seqclean tool through univec vector database (https://www.ncbi.nlm.nih.gov/tools/vecscreen/univec/). We generated a scaffold assembly of 617.76 Mb comprising 1616 contigs with an N50 size of 4.27 Mb, which was 13.7% longer than the estimated cluster bean genome size.

The long Nanopore sequencing reads were utilized to generate a de novo assembly using Canu (v1.6)^40^, which generated 3904 contigs spanning 419.66 Mb with N50 of 775 kb. This primary assembly was further polished with Illumina shotgun and Mate-Pair data using Pilon (v1.23)^41^ to generate an improved assembly of length 441.85 Mb, which comprised of 1548 contigs with N50 of 544.474 kb.

The primary assemblies from 10X supernova and Oxford Nanopore (Canu) were merged with npScarf^42^ and generated a highly contiguous assembly of 550.16 Mb comprising 1580 scaffolds with an N50 value of 7.12 Mb and longest scaffold of 35.03 Mb (Supplementary Table S2).

### Generation of linkage map

Further, an F_2_ population was developed from RGC-936/ CAZRI-15-3-8 (Supplementary Fig. S2) at Central Arid Zone Research Institute (CAZRI), Jodhpur, Rajasthan, India. A total of 142 F_2_ progenies along with both the parental genotypes were used to generate a genetic linkage map by using Genotyping by Sequencing (GBS) technology. The raw GBS reads were aligned against the genome assembly using bwa mapping software and SNP calling was done using Unified Genotyper from the Genome Analysis Toolkit GATK (v3.6)^43^. According to UGbs-Flex pipeline^44^, SNPs with allele frequencies < 0.1 and > 0.9 and adjacent SNPs were discarded. Further markers showing segregation distortion from the expected 1:2:1 Mendelian ratio were discarded on the basis of *χ*^2^ test (*p* < 0.05). Finally, we obtained 6113 markers that were imported in JoinMap (v4)^45^, program in Kyazma software package for creating the linkage map containing seven linkage groups (https://www.kyazma.nl/index.php/). Also, the linkage groups were determined at logarithm of odds (LOD) score of 6.0.

### Final assembly

A total of 1529 scaffolds containing 6113 markers in seven linkage groups were merged into specific pseudomolecules using in-house Perl script. Finally, seven genetically anchored pseudomolecules/ chromosomes along with 51 unanchored contigs resulted in the final assembly of 550.30 Mb for the cluster bean genome. The chromosome length of the cluster bean genome ranged from 61.32 Mb (Chr7) to 93.95 Mb (Chr1) with a scaffold N50 of 78.27 Mb (Fig. 1, Supplementary Tables S3, S4, and Supplementary Fig. S3).

### Assembly validation

Further, we used LTR assembly Index (LAI) to evaluate assembly quality of the cluster bean genome. LAI is a reference free genome metric that uses assembly quality of LTR-retrotransposons (LTR-RTs) to evaluate genome assembly continuity. It has been widely used to evaluate the assembly quality of repeat rich plant genomes. We calculated the LAI score of the cluster bean genome using LTR_retriever^46^ with default parameters. The results indicated that 28.44% of the cluster bean genome was occupied by LTR-RTs with LAI of 11.04. In the present study we have also calculated LAI score of other plant species including Chickpea (*Cicer arietinum*), Mungbean (*Vigna radiata*), Pigeonpea (*Cajanus cajan*), Soybean (*Glycine max*), Medicago (*Medicago truncatula*), Arabidopsis (*Arabidopsis thaliana*), and Rice (*Oryza sativa*) (Fig. 2 and Supplementary Table S4).

### Genome annotation

#### Identification and annotation of repetitive DNA sequences

For identification and annotation of repetitive DNA sequences in the cluster bean genome assembly, we used a de novo repeat library and Dfam^47^ (v3.1) database. First, RepeatModeler (v1.0.10, http://www.repeatmasker.org/RepeatModeler/) was employed to make a de novo repeat library of cluster bean genome assembly. Next, we annotated the cluster bean de novo repeat library by using Repeatmasker (v4.0.7). Then BLASTn search was performed to annotate unclassified elements from Repeatmasker with the repetitive elements in the Dfam database (https://www.dfam.org/releases/Dfam_3.1/). We identified 582,339 DNA repeat sequences covering 42.14% of the cluster bean genome. The most abundant repetitive element type was the retrotransposons making up to 29.73% of the genome, including 8.08% of LINES, 21.62% of long terminal repeats (LTRs), 23.3% of DNA transposons and 5.56% of unclassified repetitive elements (Table 2 and Supplementary Table S5).

Simple sequence repeats (SSRs) or microsatellites in the cluster bean genome were identified by MISA^48^ program with the following parameters: monomer (n ≥ 10), dimer (n ≥ 6), trimer (n ≥ 5), tetramer (n ≥ 5), pentamer (n ≥ 5), and hexamer (n ≥ 5). A total of 238,176 SSRs covering, 0.46% (2.52 Mb) of the cluster bean genome were detected. Among the SSRs, monomers were the most abundant type (71.57%), followed by dimer (16.91%), trimer (9.84%), tetramer (1.31%), pentamer (0.20%), and hexamer (0.16%), respectively (Supplementary Table S6).

#### Gene model prediction and functional annotation

From the cluster bean genome assembly, the protein-coding genes were predicted using the Seqping (version 0.1.45)^49^ pipeline. Seqping provides species-specific, unbiased gene predictions making it suitable for gene prediction of non-model plant genomes like cluster bean. Seqping pipeline processes genome and transcriptome sequences of the target species using GlimmerHMM, SNAP, and AUGUSTUS pipelines, followed by combining predictions from all three tools in association with transcriptome evidence, with MAKER2. The RNA sequencing data generated earlier by Rawal et al.^16^ was used for transcript assembly with Trinity (v 2.1.1)^50^ and the resulting transcripts were utilized in the Seqping pipeline. Initially, Seqping predicted 37,509 protein-coding genes and after clustering with CD-HIT (version 4.6)^51^, 34,680 non-redundant gene predictions were obtained (Supplementary Table S7 and Fig. S4).

The efficiency of the gene prediction and completedness was evaluated with Benchmarking Universal Single-Copy Orthologs (BUSCO) analysis, using the Plant embryophyta_odb10 lineage with Arabidopsis species (BUSCO v3.1.0)^52^ containing 1375 single copy genes. The BUSCO analysis reported 96.90% of gene predictions as complete, including 64% complete single-copy and 32.9% duplicated genes, 2.6% of missing gene models and 0.5% of fragmented gene models (Supplementary Fig. S5), indicating a high efficiency of gene prediction.

The functional annotation and Gene ontology (GO) terms for each predicted gene model were allocated via InterProScan 5 (v 5.25-64.0)^53^ and Blast2GO (v 4.1)^54^ respectively. About 28,955 (78.93%) genes were annotated successfully (Supplementary Table S8 and Fig. S6).

#### Identification and annotation of Non-coding RNA genes

Non-coding RNAs (ncRNAs) including tRNAs, rRNAs, and snRNAs in cluster bean genome were identified and annotated using various software packages and databases. First, tRNAscan-SE (version 1.3.1)^55^ with default parameters was used to identify and annotate tRNAs and their secondary structures. Total, 474 tRNA genes corresponding to a total length of 33.4 kb were identified. Further, to annotate the ribosomal RNA (rRNA) and small nuclear RNA (snRNA), BLASTn search against the Rfam database (version 14.1)^56^ was performed. We found 922 rRNA genes with a total length of 654.07 kb and 347 snRNA, with a total length of 34.8 kb. The Coding Potential Calculator 2 (CPC2)^44^ was used to predict the coding capacity of all mRNAs using default settings. The organization of cluster bean genome assembly, gene density, DNA repeat elements, SSRs and duplicate genes are shown in Fig. 1 and Supplementary Fig. S7.

### Comparative genome analysis

#### Phylogenetic analysis and estimation of divergence time

As mentioned in previous studies, the molecular comparison of Single-copy orthologs among different taxa is the most common approach to study the phylogenetics. We used OrthoMCL (v2.0.9)^57^ to identify orthologous genes among cluster bean and other important crop and model plants. Finally, we identified a total of 51 single-copy orthologs from 12 angiosperm species including *C. tetragonoloba, Phaseolus vulgaris, Vigna radiata, Cajanus cajan, Glycine max, Cicer arietinum, Medicago truncatula, Vicia faba, Arachis hypogaea, Arabidopsis thaliana* and *Nelumbo nucifera* and a monocot *Oryza sativa*. Based on this consensus orthologous gene set, a phylogenetic time-tree of the twelve plant species was constructed using MEGAX^58,59^. We used the *A. thaliana* and *O. sativa* divergence time (152 MYA) and the monocot and eudicot divergence time (115-308 MYA)^60^ as calibrators.

#### Reconstruction of ancestral legume karyotype

We adapted the ancestral genome reconstruction method described in Ren et al.^34^ to reconstruct the ancestral legume karyotype^34^. For this study, we used genome assemblies and annotations of *Arachis ipaensis*^61^*, Arachis duranensis*^61^*,* and *Arachis hypogaea*^62^ from PeanutBase (https://www.peanutbase.org/), *Phaseolus vulgaris*^63^*, Lupinus angustifolius*^64^*, Cicer arietinum*^65^*, Cyamopsis tetragonoloba, Glycine max*^66^*, Vigna radiata*^67^*, Medicago truncatula*^68^*,* and *Cajanus cajan*^69,70^ from legume information system (https://legumeinfo.org/). Protein sequences were used to perform the synteny analysis between *C. tetragonoloba* and each of the other selected species using DIAMOND protein aligner with default parameter^71^. Syntenic blocks were then constructed from the orthologous groups using the DAGchainer program^72^. The output syntenic blocks from DAGchainer were manually checked and overlapping syntenic blocks were removed. First, the syntenic blocks were broken down into just gene pairs by removing the block information lines for the comparison between *C. tetragonoloba* and one of the selected species. The start and end gene pairs of each syntenic block were marked using unique symbols to distinguish start and end of each block. Then, the syntenic blocks between *C. tetragonoloba* and one of the remaining species were added to the existing file using the gene pairs within those syntenic blocks as anchors. The newly defined syntenic blocks were used as syntenic block markers across all selected species. Syntenic-block markers with fewer than 4 genes in them were discarded. Thus, the output syntenic blocks from DAGchainer were used as “markers” to specify the shared features among the selected genomes using the scripts provided in Ren et al.^34^. An input file was generated based on the markers and their orientations in each selected species. The MLGO web server (http://www.geneorder.org/server.php) was used to reconstruct the ancestral genomes of selected legume species^73^.

#### Gene Family analysis

The orthologous genes and phylogenetic relationship inferred from OrthoMCL analysis were subjected to CAFÉ v4.2^74^ to analyze changes in gene family size during evolution. The CAFÉ program uses a random birth and death model to predict the size of each gene family including significant (*p*-values) gene gain and loss across a user-specified phylogenetic tree.

#### Whole Genome duplication analysis

For identifying WGD in Guar genomes, we used the distribution of synonymous substitutions/site (dS) between paralogous gene pairs across the entire genome. We used the entire genome to extract dS estimates for better compare dS distributions*.* We used WGDdetector^75^, which estimates dS distributions across gene families to correct for redundant dS values among paralogs, with Mmseqs2 as the cluster engine with e-value 1e-10. Gene families were then built using Markov clustering algorithm using MCL. For each gene family, a protein alignment was constructed using MAFFT and PAL2NAL. This alignment was used as a guide for aligning the DNA sequences of gene family pairs. Only gene pairs with a gap-stripped alignment length > 90 bp were considered for further analyses. Gene families were subdivided into subfamilies for which dS estimates between genes did not exceed a value of 5. To correct for the redundancy of dS values (a gene family of n members produces n[n–1]/2 pairwise dS estimates for n − 1 retained duplication events), an average linkage clustering approach was used. Briefly, for each gene family, a tentative phylogenetic tree was constructed by average linkage hierarchical clustering, using dS as a distance measure. Finally, histogram plot was constructed using Rscript with all the dS values of each subfamily. We also used SyMAP tool (SyMAP v5.0.6)^76^ to perform self-synteny in the cluster bean genome for the identification of duplicated regions.

#### Transcriptome analysis

To validate genome annotation and understand transcriptional landscape of galactomannan biosynthesis, we performed transcriptome analysis of six different cluster bean tissues including previously reported data (Shoot, Leaf, Flower)^16^ and RNA-seq data generated from three stages of pod development (25-DAF, 39-DAF, and 50-DAF) of RGC-936 cultivar (BioProject: PRJNA545776)^77^. A total of 288.2 million reads from six different cluster bean tissues were used for transcriptome analysis (Supplementary Table S10). First sequencing quality of all the raw RNA-seq reads was analyzed with FastQC software (http://www.bioinformatics.babraham.ac.uk/projects/fastqc/). All the raw reads were subjected to Cutadapt software (https://www.bioinformatics.babraham.ac.uk/projects/trim_galore/) for primary quality control and adapter removal. All the cleaned reads with Q ≥ 20 were used for cluster bean reference genome based transcriptome analysis. For RNA-seq analysis we followed stand-alone pipeline as described by Pertea et al.^78^ involving Hisat2^79^, Stringtie^80^, and Ballgown^81^ software. Hisat2 was used to align high-quality RNA-seq reads against cluster bean genome, followed by transcript assembly and estimation of transcript abundance using StringTie and Ballgown. About 67.86–89.48% of the reads mapped to cluster bean genome indicating high-quality of the genome assembly (Supplementary Table S10).

### Ethical approval

This article contains no studies with human participants conducted by any of the authors.

## Supplementary Information

Supplementary Information 1.

Supplementary Information 2.

## Data Availability

The Guar (*Cyamopsis tetragonoloba*) Genome sequencing data generated and/or analysed during the current study are available in NCBI under BioProject: PRJNA630418.
