# Supplementary material for: The chromosome-scale genome assembly of cluster bean provides molecular insight into edible gum (galactomannan) biosynthesis family genes
Source: Sci Rep. 2023 Jun 19;13:9941. doi: 10.1038/s41598-023-33762-3 (PMC10279686; doi:10.1038/s41598-023-33762-3)
Supplement: Supplementary file 1 — Supplementary Information 1. [file 41598_2023_33762_MOESM1_ESM.pptx]

## Slide 1
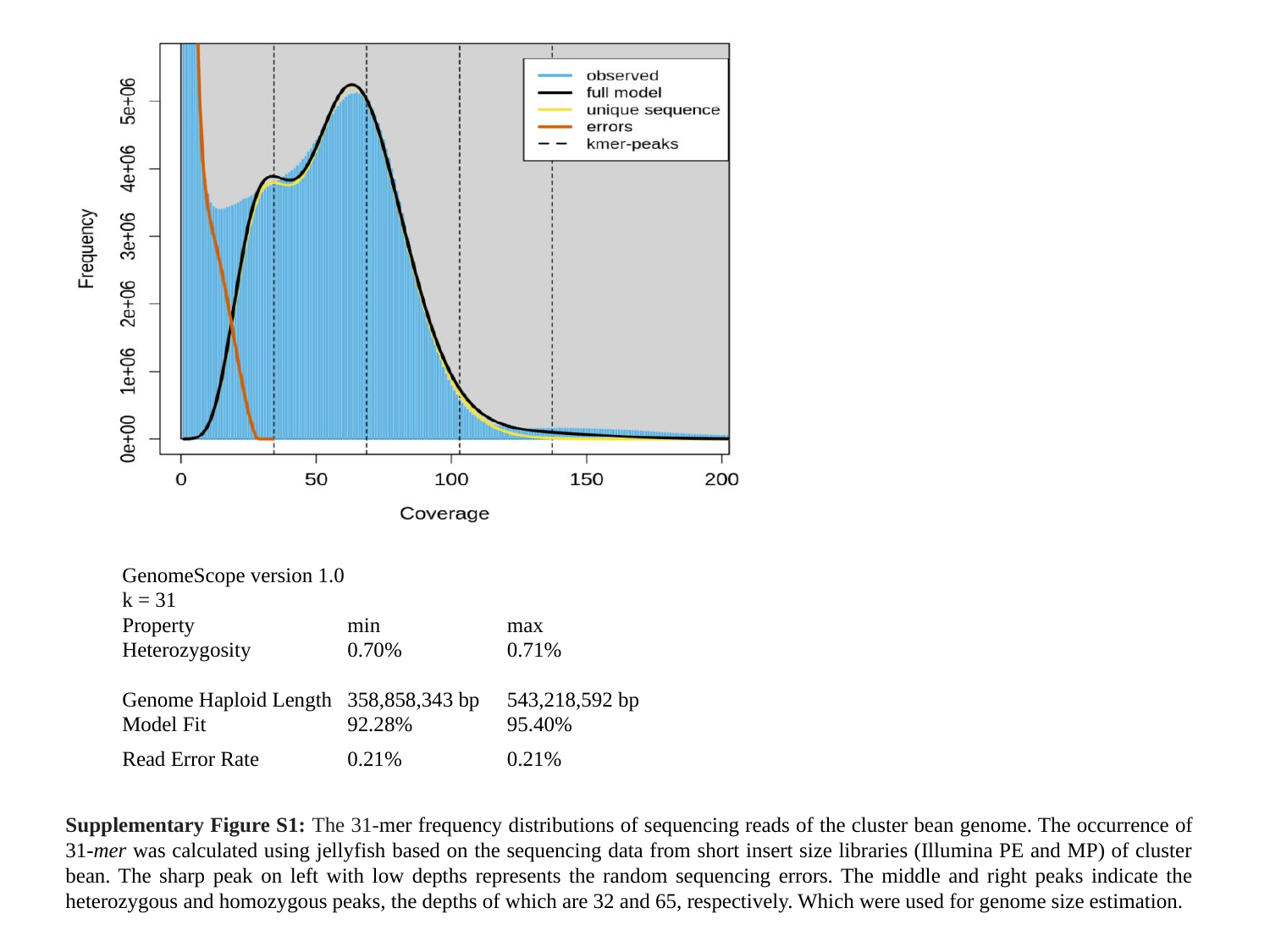

| GenomeScope version 1.0 | | |
| --- | --- | --- |
| k = 31 | | |
| Property | min | max |
| Heterozygosity | 0.70% | 0.71% |
| Genome Haploid Length | 358,858,343 bp | 543,218,592 bp |
| Model Fit | 92.28% | 95.40% |
| Read Error Rate | 0.21% | 0.21% |
Supplementary Figure S1: The 31-mer frequency distributions of sequencing reads of the cluster bean genome. The occurrence of 31-mer was calculated using jellyfish based on the sequencing data from short insert size libraries (Illumina PE and MP) of cluster bean. The sharp peak on left with low depths represents the random sequencing errors. The middle and right peaks indicate the heterozygous and homozygous peaks, the depths of which are 32 and 65, respectively. Which were used for genome size estimation.

## Slide 2
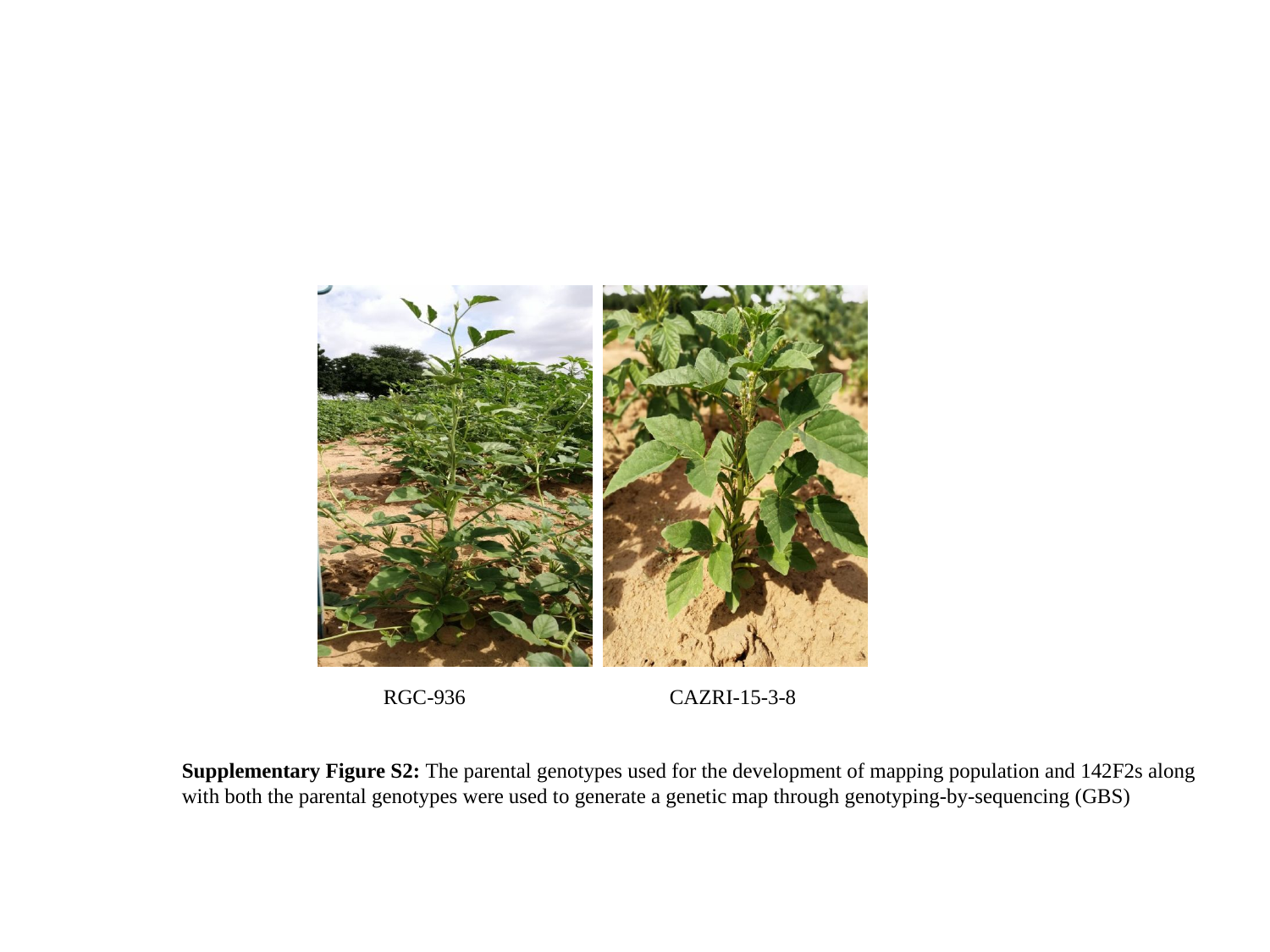

RGC-936
CAZRI-15-3-8
Supplementary Figure S2: The parental genotypes used for the development of mapping population and 142F2s along with both the parental genotypes were used to generate a genetic map through genotyping-by-sequencing (GBS)

## Slide 3
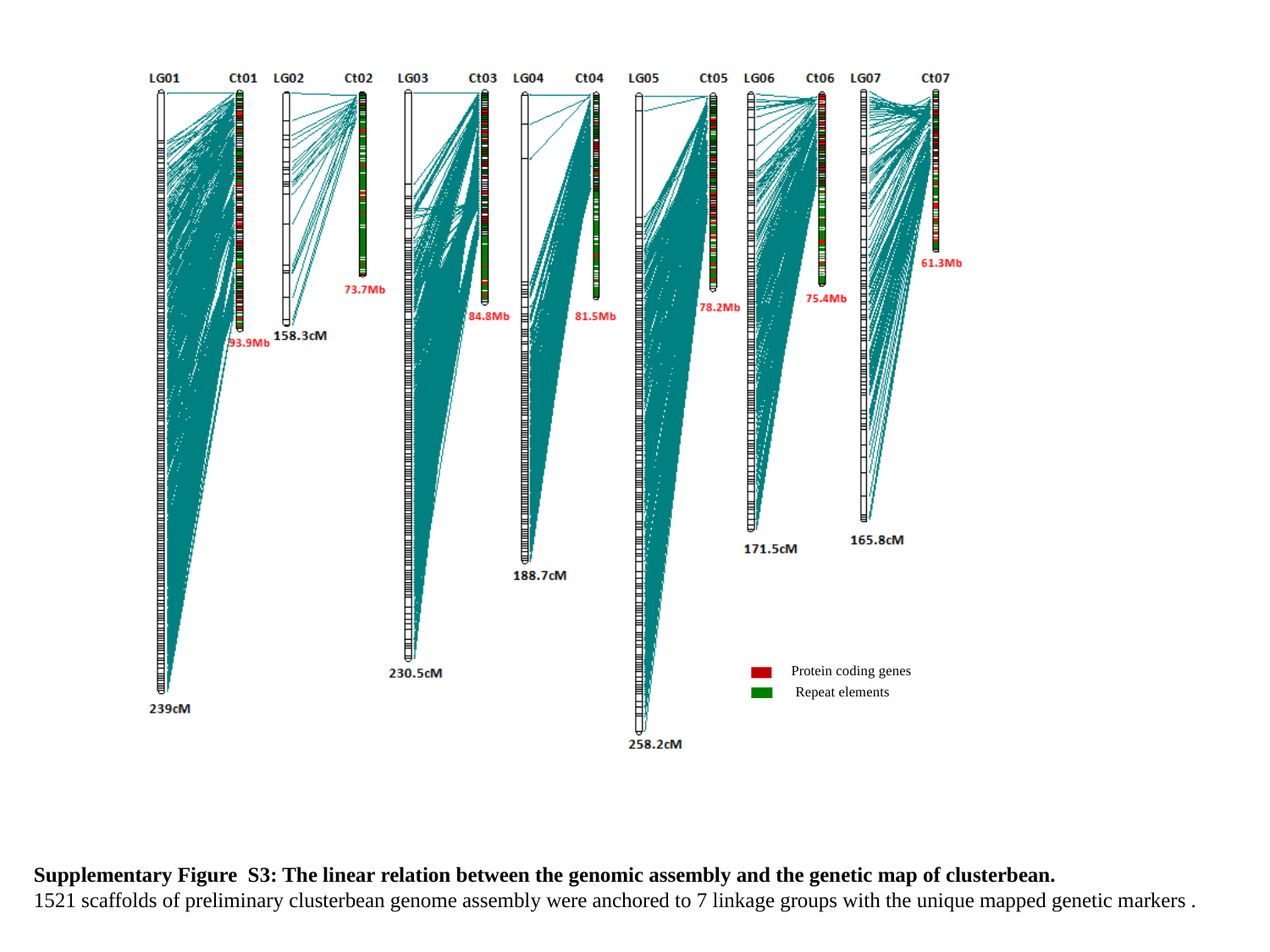

Protein coding genes
Repeat elements
Supplementary Figure S3: The linear relation between the genomic assembly and the genetic map of clusterbean.
1521 scaffolds of preliminary clusterbean genome assembly were anchored to 7 linkage groups with the unique mapped genetic markers .

## Slide 4
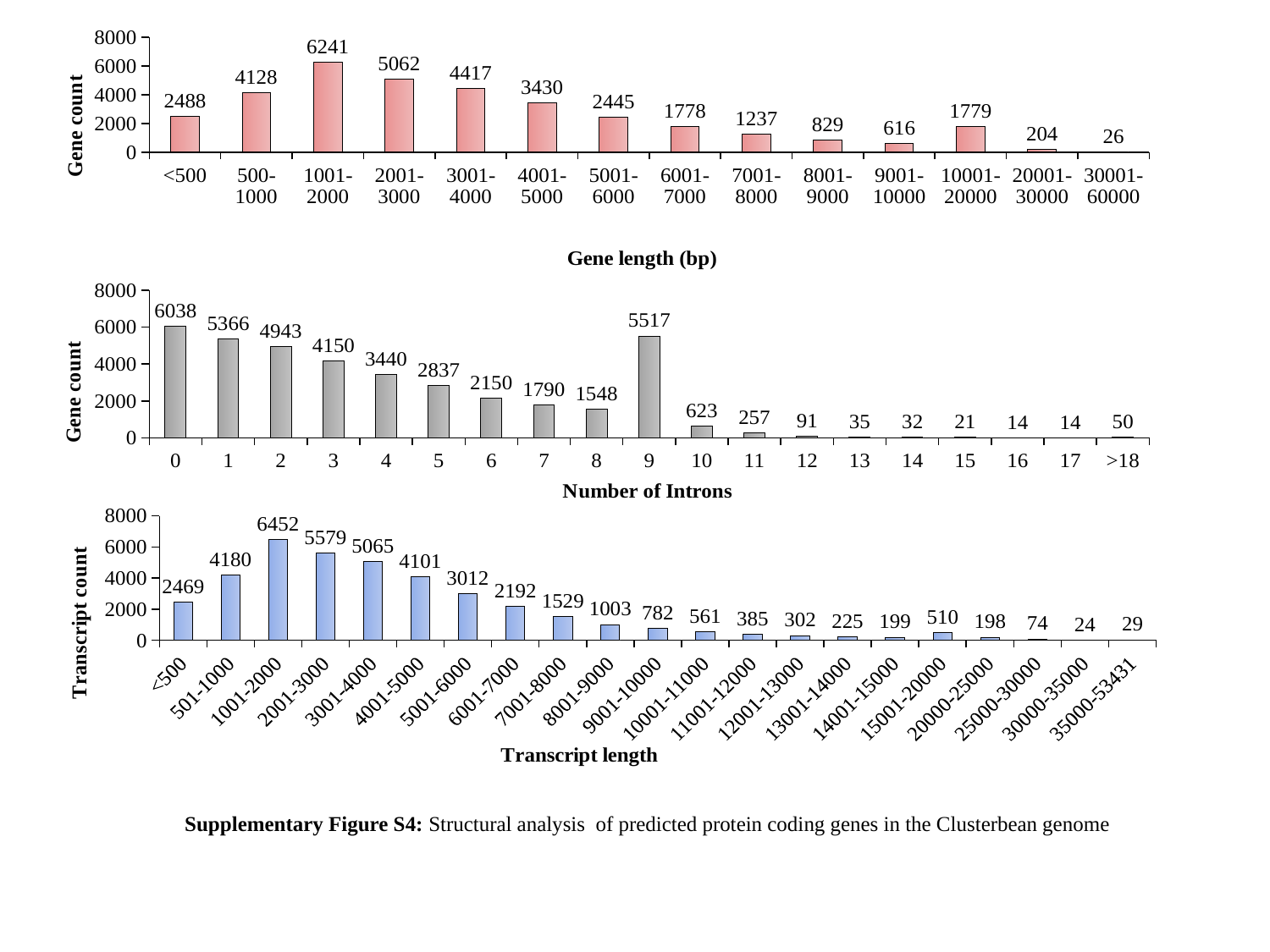

### Chart
| Category | Gene Count |
|---|---|
| <500 | 2488.0 |
| 500-1000 | 4128.0 |
| 1001-2000 | 6241.0 |
| 2001-3000 | 5062.0 |
| 3001-4000 | 4417.0 |
| 4001-5000 | 3430.0 |
| 5001-6000 | 2445.0 |
| 6001-7000 | 1778.0 |
| 7001-8000 | 1237.0 |
| 8001-9000 | 829.0 |
| 9001-10000 | 616.0 |
| 10001-20000 | 1779.0 |
| 20001-30000 | 204.0 |
| 30001-60000 | 26.0 |
### Chart
| Category | Gene Count |
|---|---|
| 0 | 6038.0 |
| 1 | 5366.0 |
| 2 | 4943.0 |
| 3 | 4150.0 |
| 4 | 3440.0 |
| 5 | 2837.0 |
| 6 | 2150.0 |
| 7 | 1790.0 |
| 8 | 1548.0 |
| 9 | 5517.0 |
| 10 | 623.0 |
| 11 | 257.0 |
| 12 | 91.0 |
| 13 | 35.0 |
| 14 | 32.0 |
| 15 | 21.0 |
| 16 | 14.0 |
| 17 | 14.0 |
| >18 | 50.0 |
### Chart
| Category | Transcript Count |
|---|---|
| <500 | 2469.0 |
| 501-1000 | 4180.0 |
| 1001-2000 | 6452.0 |
| 2001-3000 | 5579.0 |
| 3001-4000 | 5065.0 |
| 4001-5000 | 4101.0 |
| 5001-6000 | 3012.0 |
| 6001-7000 | 2192.0 |
| 7001-8000 | 1529.0 |
| 8001-9000 | 1003.0 |
| 9001-10000 | 782.0 |
| 10001-11000 | 561.0 |
| 11001-12000 | 385.0 |
| 12001-13000 | 302.0 |
| 13001-14000 | 225.0 |
| 14001-15000 | 199.0 |
| 15001-20000 | 510.0 |
| 20000-25000 | 198.0 |
| 25000-30000 | 74.0 |
| 30000-35000 | 24.0 |
| 35000-53431 | 29.0 |Supplementary Figure S4: Structural analysis of predicted protein coding genes in the Clusterbean genome

## Slide 5
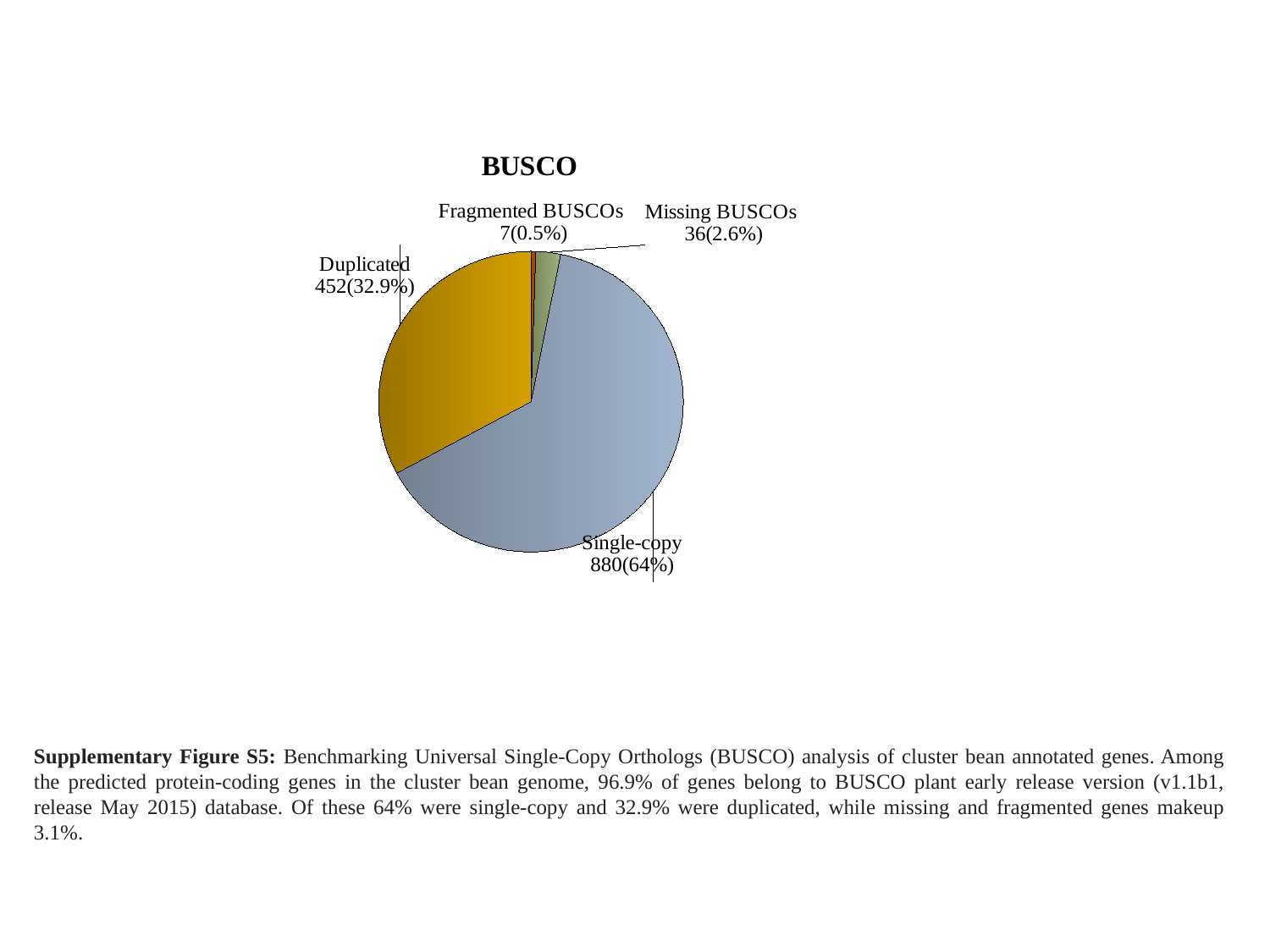

[unsupported chart]
Supplementary Figure S5: Benchmarking Universal Single-Copy Orthologs (BUSCO) analysis of cluster bean annotated genes. Among the predicted protein-coding genes in the cluster bean genome, 96.9% of genes belong to BUSCO plant early release version (v1.1b1, release May 2015) database. Of these 64% were single-copy and 32.9% were duplicated, while missing and fragmented genes makeup 3.1%.

## Slide 6
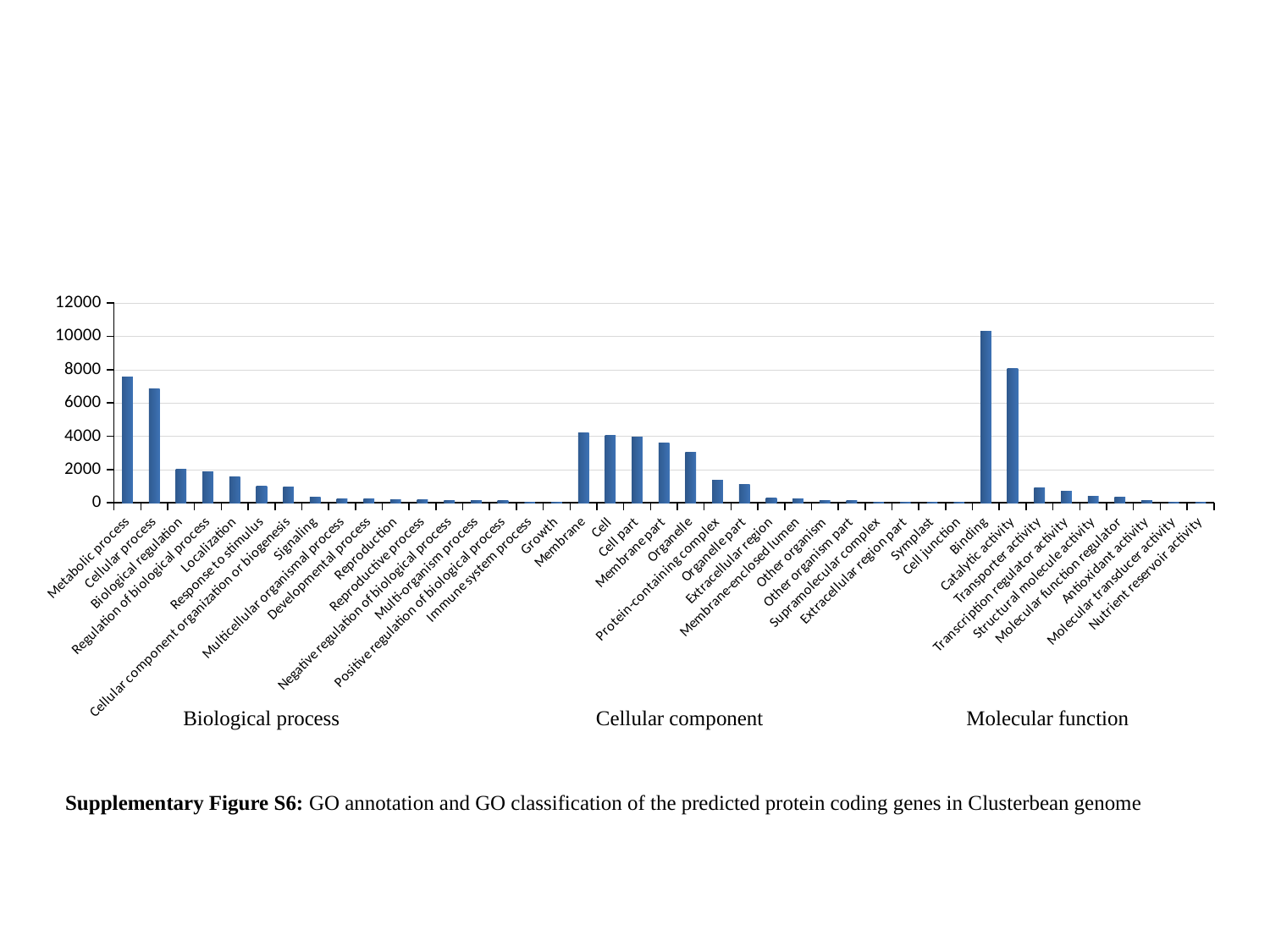

### Chart
| Category | Number of genes |
|---|---|
| Metabolic process | 7568.0 |
| Cellular process | 6824.0 |
| Biological regulation | 2001.0 |
| Regulation of biological process | 1846.0 |
| Localization | 1574.0 |
| Response to stimulus | 1012.0 |
| Cellular component organization or biogenesis | 926.0 |
| Signaling | 321.0 |
| Multicellular organismal process | 238.0 |
| Developmental process | 233.0 |
| Reproduction | 173.0 |
| Reproductive process | 172.0 |
| Negative regulation of biological process | 142.0 |
| Multi-organism process | 140.0 |
| Positive regulation of biological process | 130.0 |
| Immune system process | 36.0 |
| Growth | 20.0 |
| Membrane | 4208.0 |
| Cell | 4073.0 |
| Cell part | 3934.0 |
| Membrane part | 3605.0 |
| Organelle | 3046.0 |
| Protein-containing complex | 1371.0 |
| Organelle part | 1107.0 |
| Extracellular region | 272.0 |
| Membrane-enclosed lumen | 235.0 |
| Other organism | 159.0 |
| Other organism part | 159.0 |
| Supramolecular complex | 60.0 |
| Extracellular region part | 38.0 |
| Symplast | 25.0 |
| Cell junction | 25.0 |
| Binding | 10311.0 |
| Catalytic activity | 8049.0 |
| Transporter activity | 895.0 |
| Transcription regulator activity | 670.0 |
| Structural molecule activity | 377.0 |
| Molecular function regulator | 327.0 |
| Antioxidant activity | 112.0 |
| Molecular transducer activity | 55.0 |
| Nutrient reservoir activity | 47.0 |Biological process
Cellular component
Molecular function
Supplementary Figure S6: GO annotation and GO classification of the predicted protein coding genes in Clusterbean genome

## Slide 7
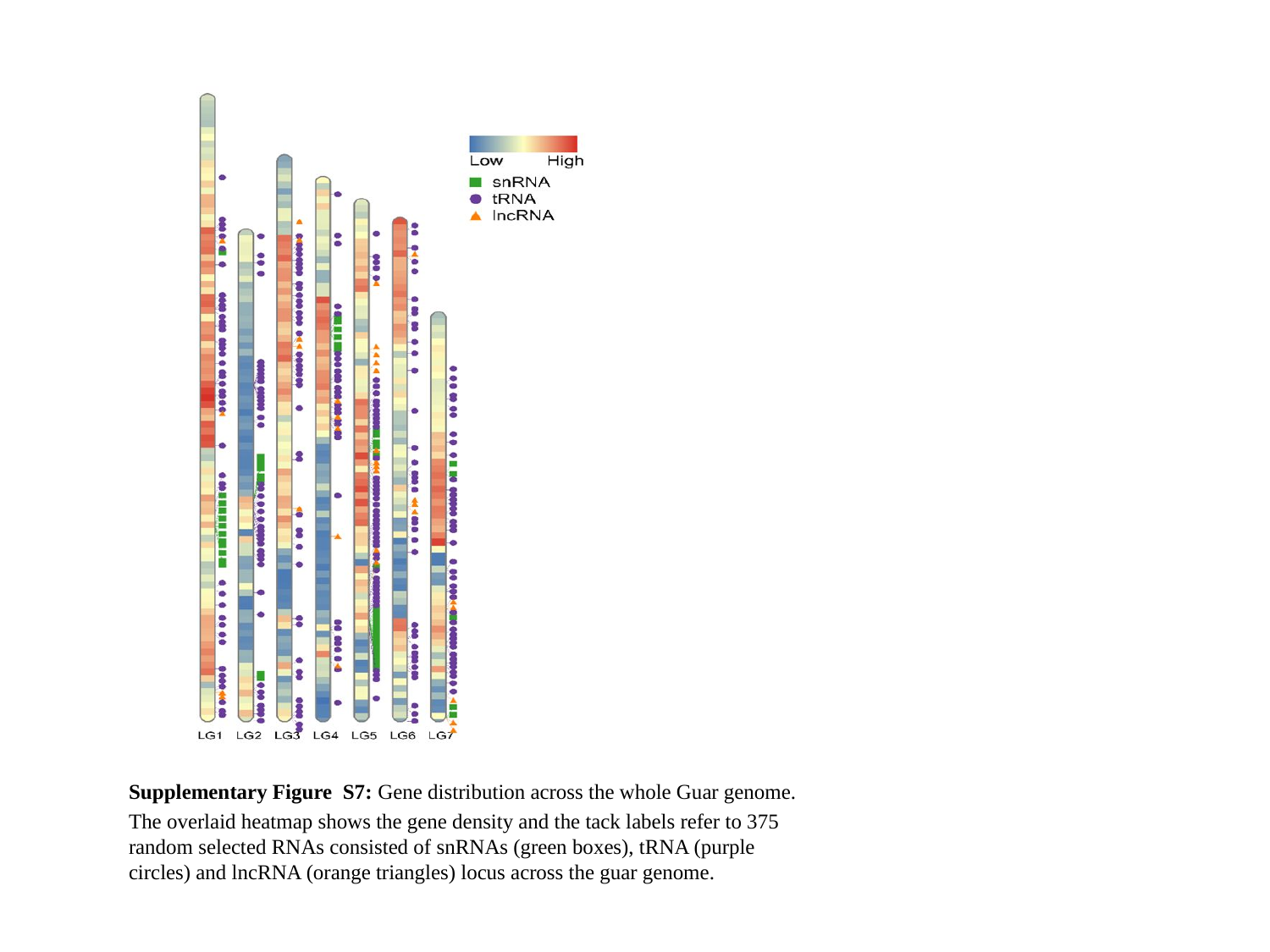

Supplementary Figure S7: Gene distribution across the whole Guar genome.
The overlaid heatmap shows the gene density and the tack labels refer to 375 random selected RNAs consisted of snRNAs (green boxes), tRNA (purple circles) and lncRNA (orange triangles) locus across the guar genome.
